# Leveraging Genomic Data to Examine the Causal Impact of Alcohol, Tobacco, Cannabis, and Opioid Use on Biological and Cognitive Ageing

**DOI:** 10.1111/adb.70066

**Published:** 2025-07-13

**Authors:** Jared V. Balbona, Paul Jeffries, Aaron J. Gorelik, Elliot C. Nelson, Ryan Bogdan, Arpana Agrawal, Emma C. Johnson

**Affiliations:** ^1^ Department of Psychiatry, School of Medicine Washington University in St. Louis St. Louis Missouri USA; ^2^ Department of Psychological and Brain Sciences Washington University in St. Louis St. Louis Missouri USA

**Keywords:** ageing, behavioural genetics, causal inference, GWAS, Mendelian randomization, substance use, whole genome methods

## Abstract

Although substance use is associated with a shortened lifespan, impeded health and accelerated biological ageing, the factors contributing to the associations between substance use and ageing are poorly understood. We used summary statistics from genome‐wide association studies (GWAS) to investigate whether substance involvement (*N* from 28K to 2M)—including alcohol, tobacco, cannabis and opioid use and use disorders—is genetically correlated with various ageing metrics (*N* from 162K to 2.7M) and whether these correlations reflect shared genetic etiologies or putative causal relationships. Using Linkage Disequilibrium Score Regression (LDSC), we found widespread evidence of genetic correlations between substance use/use disorders and indices of physical, cognitive and biological ageing. We then employed a series of Mendelian randomization–based approaches, finding significant causal effects of genetic predispositions to both tobacco use disorder and quantity of tobacco smoked on various markers of ageing. Causal effects of problematic alcohol use and cannabis use disorder were also found, though findings were mixed. Evidence of reverse causality (i.e., ageing causing substance use), meanwhile, was scant. Collectively, these results demonstrate strong triangulation across approaches and highlight the importance of integrating genetic insights into public health strategies for reducing the burden of SUDs across the lifespan.

## Abstract 2

Prior studies have linked substance use to adverse ageing outcomes, but it remains unclear whether these associations reflect causal effects, shared genetic influences or confounding. We found widespread genetic correlations between substance use/use disorders and various ageing metrics. A series of Mendelian randomisation analyses supported causal effects of substance use—particularly tobacco and, to a lesser extent, alcohol and cannabis use—on ageing, with little evidence of reverse causality (i.e. ageing causing substance use).

## 1 Introduction

Chronic substance use and substance use disorders (SUDs)—psychiatric conditions marked by interpersonal, physiological and psychological problems resulting from the loss of control over substance use—are among the leading causes of preventable death globally, placing a substantial burden on both individuals and society [1]. Despite this, not all individuals who use substances or have SUDs suffer premature mortality as a result. Instead, many experience recovery and remission, whereas others continue using substances maladaptively into later life, as evidenced by rising SUD rates among older adults [2]. As global life expectancy and substance availability have increased [2], it is crucial to understand if and how substance involvement directly complicates ageing and health across the lifespan.

Psychoactive substances can directly and indirectly impact physical health, with evidence linking prolonged substance exposure to discrepancies between individuals' chronological age (i.e., the number of years that an individual has lived) and their biological age (which reflects their level of accumulated cellular/molecular damage and consequent neurophysiological functioning) [3]. What is less well understood, however, is the extent to which substance involvement *causally* modifies physical and biological ageing (e.g., via neurotoxic effects) as opposed to both SUDs and accelerated ageing resulting from shared predisposing factors (e.g., genetic variants and neighbourhood disadvantage). Given the heritable nature of substance use [4] and most ageing metrics [5], genetically informed study designs are well suited to estimate the extent of shared genetic vulnerability to both domains; these methods can then subsequently determine whether their genetic overlap is due to shared aetiology, shared confounding effects or a causal relationship between substance use and ageing [6].

In the present study, we examined the relationship between eight substance‐related traits and eight ageing‐related traits (see Table S1). These ageing traits included Alzheimer Disease diagnosis, frailty index, healthspan, parental lifespan, GrimAge, leukocyte telomere length, brain‐age gap and a multivariate factor capturing shared variance across ageing traits (mvAge). Note that these measures capture a broad, lifespan‐oriented perspective on ‘ageing’ that includes not only geriatric health outcomes (e.g., frailty) but also indicators of biological age that occur throughout an individual's life (e.g., telomere length). Similarly, we are not only examining SUDs and addictions—including problematic alcohol use, tobacco use disorder, opioid use disorder, cannabis use disorder and a multivariate factor capturing shared variance across SUDs (mvSUD)—but also levels of substance consumption that do not necessarily meet the criteria for a disorder/addiction: drinks per week, smoking initiation (smoking ≥ 100 times) and lifetime cannabis ever use. In doing so, we are seeking to capture variation in substance involvement beyond clinical diagnostic thresholds.

We first assessed the genetic overlap between these substance use and ageing traits by estimating their genetic correlations with Linkage Disequilibrium Score Regression (LDSC) [7]. Genetic correlations quantify the extent to which the genetic factors associated with one trait are also associated with another, which may indicate shared biological aetiology, a common underlying confounder or a potential causal relationship [8, 9]. Such results are meaningful in and of themselves, as they provide insight into whether the previously observed phenotypic associations between substance use and ageing traits may have a genetic basis. Additionally, genetic correlation testing also serves as a key prerequisite for our downstream causal analyses, as a lack of shared genetic variation between two traits would indicate a lack of sufficient power to detect causal effects.

**FIGURE 1:**
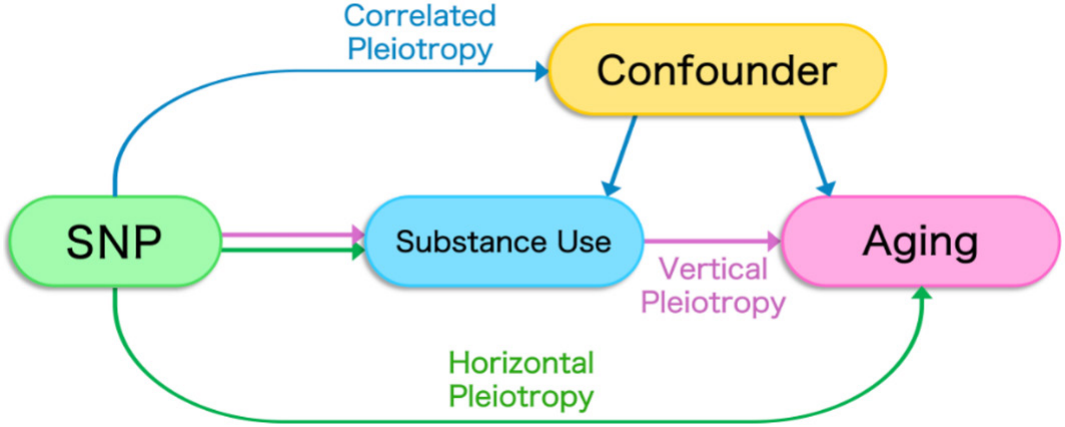
Vertical pleiotropy occurs when a single nucleotide polymorphism (SNP) influences multiple traits through a direct causal pathway, where the SNP affects one trait that in turn affects another. In contrast, horizontal pleiotropy happens when a single SNP independently influences multiple traits through separate and distinct mechanisms, without one trait affecting the other. Correlated pleiotropy involves a situation where a single gene affects multiple traits that are correlated, often because the traits share common genetic or environmental influences.

More specifically, genetic overlap between two traits can result from several different processes, including (1) one trait having a direct causal effect on a second trait (a phenomenon known as ‘vertical pleiotropy’); (2) the two otherwise independent traits both being influenced by a common genetic variant (‘horizontal pleiotropy’); and (3) genetic variants being associated with a shared confounding factor for both traits (‘correlated pleiotropy’; Figure 1) [9]. To tease apart these distinct mechanisms, we examined each significantly genetically correlated trait pair using Mendelian randomization (MR)—a genetically informed approach used to determine whether the relationship between two traits is likely to be causal [10, 11]. Briefly, MR leverages the quasirandom assortment of genes from parents to offspring in order to estimate the direct causal effect of an exposure (e.g., substance use) on an outcome (e.g., ageing).

The random segregation of alleles during meiosis ensures that, on average, the inheritance of a particular genetic variant is independent of traits that the variant does not causally affect; in this way, the random assortment of genes serves as an analogue to a randomized control trial, in which lifestyle and environmental confounders are randomly distributed across the ‘treatment’ and ‘control’ groups—those who do and do not have a copy of the effect allele, respectively [12]. As such, MR allows researchers to infer causality in situations where shared genetic variation is expected and randomized control trials are not feasible.

To strengthen causal inference and account for potential biases, we employed multiple MR approaches, each with different assumptions and strengths (Table S1). Following recent recommendations [13], inverse‐variance weighted MR (IVW‐MR) with multiplicative random effects [14] served as our primary analysis, as it has the greatest statistical power to detect causal effects. We further bolstered these analyses by also incorporating MR‐Egger [15], median weighted MR [16], MR‐Pleiotropy RESidual Sum and Outlier (MR‐PRESSO) [17] and Causal Analysis Using Summary Effect Estimates (CAUSE) [18] approaches (see Section 2). Triangulation across methods strengthens causal inference, providing a richer and more comprehensive view of the interplay between substance use and ageing that may support the development of targeted public health interventions and mitigate the impact of substance use on ageing and long‐term health [19].

## 2 Methods

### 2.1 Data Sources and Phenotypes

All genetic data used were taken from genome‐wide association studies (GWASs) summary statistics. Briefly, GWASs are large‐scale, hypothesis‐free studies that scan the genome to identify genetic variants associated with a particular trait or disease; the summary statistics for each GWAS thus contain the degree of association for each genetic variant with the outcome of interest. For the present study, we prioritized traits with established or hypothesized substance use—ageing associations. Additionally, although there was no strict threshold for inclusion, we sought to prioritize traits with well‐powered, publicly available GWAS summary statistics that contain robust genome‐wide significant hits (i.e., traits with significant heritability), ensuring sufficient statistical power for genetic correlation and MR analyses. Furthermore, to minimize bias due to ancestry/population stratification (a major concern in GWAS‐based research [20]), all summary statistics used were derived in individuals of European descent (Table S2).

We selected substance use phenotypes that reflect both clinically defined SUDs as well as subthreshold measures of substance consumption. Specifically, we examined problematic alcohol use [21] (*N* = 903 147), drinks per week [22] (*N* = 2 669 029), smoking initiation [22] (*N* = 2 669 029), tobacco use disorder [23] (*N* = 739 895), cannabis ever use [24] (*N* = 162 082), cannabis use disorder [25] (*N* = 886 025), opioid use disorder [26] (*N* = 425 994) and a multivariate substance use factor (mvSUD; *N* = 1 025 550) [27]. As previously mentioned, traits were included in part based on the availability of well‐powered GWAS datasets, such that other potentially relevant phenotypes (e.g., cocaine use and use disorder) were not examined due to concerns regarding statistical power.

For ageing‐related phenotypes, we examined Alzheimer disease diagnosis (*N* = 472 174) [28], frailty index [29] (accumulated health deficits over time; *N* = 175 226), healthspan [30] (the number of years lived in good health; *N* = 300 447), parental lifespan [31] (a proxy for the proband's lifespan; *N* = 1 012 240), GrimAge [32] (an epigenetic biomarker predictive of mortality risk; *N* = 34 710), leukocyte telomere length [33] (a cellular marker of biological ageing; *N* = 472 174), brain‐age gap [34] (the difference between MRI‐predicted and chronological brain age; *N* = 28 104) and mvAge [35] (a multivariate factor capturing shared variance across ageing traits; *N* = 1 958 774). Although not an exhaustive list of all ageing‐relevant traits, our aim was to include a sufficiently broad spectrum of measures that encompass both clinical outcomes as well as continuous measures of biological ageing across the lifespan. Finally, note that although some of these ageing traits (i.e., Alzheimer disease, frailty index, brain age gap and GrimAge) reflect *increased* ageing, others (mvAge, parental lifespan, healthspan and telomere length) reflect *decreased* ageing; thus, higher values in the latter group of traits are indicative of particularly healthy (rather than accelerated) ageing.

### 2.2 Study Design

We first assessed the genetic overlap between all pairwise combinations of substance use and ageing traits using Linkage Disequilibrium Score Regression (LDSC), which estimates genetic correlations using GWAS summary statistics. After identifying trait pairs with significant genetic correlations—which may be indicative of potential biological links between them—we conducted MR analyses to identify potential causal relationships among the genetically correlated pairs.

### 2.3 Linkage Disequilibrium Score (LDSC) Regression

To assess the degree of genetic overlap between traits, we applied LDSC [7] to all pairs of traits, both within‐domain (i.e., ageing–ageing and SUD–SUD) and between‐domain (SUD–ageing). LDSC is a statistical method that leverages GWAS summary statistics and patterns of linkage disequilibrium (i.e., the nonrandom association of genetic variants with one another) to quantify the degree of shared genetic overlap between two traits. Importantly, LDSC estimates are not biased by sample overlap, making it a highly effective method for use with multiple potentially overlapping datasets. Prior to analysis, each trait's summary statistics were munged using LDSC. All included single nucleotide polymorphisms (SNPs) were nonpalindromic, autosomal and genome‐wide significant (*p* ≤ 5 × 10^−8^). To exclude variants adjacent to the variants of interest, SNPs were clumped based on their linkage disequilibrium (*r* ^2^ = 0.01; *p* < 5 × 10^−3^) within a genomic window of 10 kb, using the 1‐kG European linkage disequilibrium estimates as our reference panel [36]. Additionally, each effect allele was aligned to the human genome reference sequence (GRCh37) to ensure that the effect of a given SNP corresponds to the same allele for both the exposure and the outcome. Because several of our traits are nonindependent of one another (e.g., problematic alcohol use and drinks per week), a false discovery rate (FDR)‐corrected *p*‐value threshold of 0.05 was employed to reduce the risk of Type I errors.

### 2.4 Mendelian Randomization Methods

A valid genetic instrument for traditional MR‐based approaches is defined by three key assumptions: (1) It is associated with the exposure/risk factor; (2) it does not share a common cause with the outcome (e.g., population stratification); and (3) it only influences the outcome through the exposure of interest [37]. These assumptions are often violated when a genetic instrument independently affects both the exposure and outcome (horizontal pleiotropy) or when it is associated with a confounding pathway (correlated pleiotropy) [18]. Thus, the present study employs a multipronged MR approach, incorporating several complementary MR‐based methods that each make different assumptions about the nature of the instrument‐exposure‐outcome relationships, thereby increasing the robustness of our causal inferences.

All approaches used in the present study are two‐sample MR approaches, which allow the GWAS discovery samples used for the exposure and outcome to be drawn from different populations [38]. In line with recent recommendations [13], inverse‐variance weighted MR (IVW‐MR) with multiplicative random effects [14] served as our primary analysis. IVW‐MR is not only the most efficient MR method, but it is also able to account for heterogeneity in the variants' causal estimates, making it the most widely used MR method when incorporating multiple instruments. However, IVW‐MR requires the assumption that all variants are valid, as well as the assumption that the pleiotropic effects on the outcome are randomly distributed (i.e., ‘balanced pleiotropy’) and thus do not result in any systematic bias—two assumptions that are not required by other MR approaches. Thus, our IVW‐MR analyses were then complemented by MR‐Egger regression [15], median‐weighted MR [16], Causal Analysis Using Summary Effect Estimates [18] (CAUSE) and Mendelian Randomization Pleiotropy RESidual Sum and Outlier [17] (MR‐PRESSO). Unlike IVW‐MR, MR‐Egger can allow for directional pleiotropy (rather than strictly balanced pleiotropy), whereas median‐weighted MR does not require that all instrumental variables be valid. MR‐PRESSO further augments these approaches by detecting and removing SNPs with evidence of horizontal pleiotropy, thus minimizing bias in its estimate of SNP‐based vertical pleiotropy. Finally, CAUSE is a Bayesian framework that assumes that genetic relationships between traits are a mixture of both uncorrelated and correlated horizontal pleiotropy and thus evaluates the relative contributions of both. These distinct methods collectively strengthen our conclusions by providing robustness to different types of bias and violations of MR assumptions (Table S1). To further ensure the validity of our MR results, we calculated an *I* ^2^ statistic for each test to evaluate the degree of regression dilution bias, ensuring that weak instrument bias did not unduly influence pleiotropy‐corrected estimates. Additionally, Cochran's *Q* and leave‐one‐out analyses were utilized to bolster our causal inferences.

IVW‐MR, MR‐Egger, median weighted MR, Cochran's *Q* and the leave‐one‐out analyses were all implemented using the TwoSampleMR R package (https://github.com/MRCIEU/TwoSampleMR), each with the default parameters. MR‐PRESSO was conducted using the MRPRESSO R Package Version 1.0 (https://github.com/rondolab/MR‐PRESSO) with 1000 bootstrap replications and an outlier significance threshold of 0.05. Additionally, we performed CAUSE using the R Package Cause Version 1.2.0 (https://github.com/jean997/cause). CAUSE assumes that genetic relationships between traits are a mixture of both correlated and uncorrelated pleiotropy and thus evaluates the relative contributions of both. All code used for analyses is publicly available on our online repository (https://github.com/JaredBalbona/sud_aging_pleiotropy).

## 3 Results

### 3.1 Genetic Correlations

**FIGURE 2:**
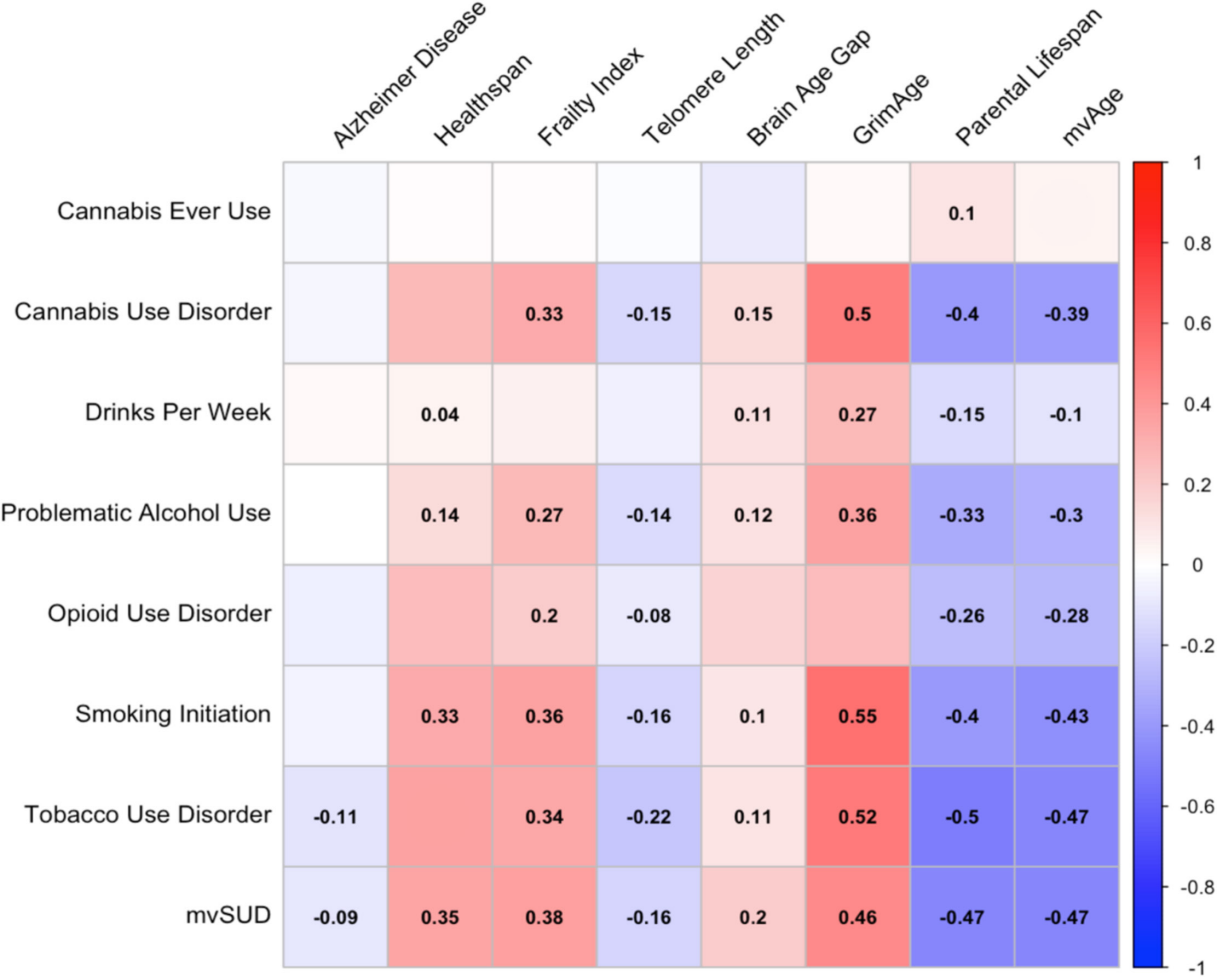
Genome‐wide genetic correlations between trait pairs, calculated using LDSC. Coefficients are provided for correlations reaching FDR‐adjusted significance (all correlations are available in Table S2). As shown, correlations were widespread, particularly for smoking and alcohol phenotypes, parental lifespan, mvSUD and mvAge.

LDSC revealed widespread correlations between substance involvement and ageing phenotypes after FDR correction, with a median absolute genetic correlation (|${\mathrm{med}}_{\mathrm{rg}}$|) of 0.18 across all between‐category trait pairs (Figure 2 and Table S3). In particular, the multivariate substance use factor (mvSUD) was significantly associated with all ageing indices (|${\mathrm{med}}_{\mathrm{rg}}$| = 0.36), with the strongest ageing associations observed for mvAge (*r*_*g*_ = −0.47, SE = 0.03, *p*_FDR_ < 0.001), parental lifespan (*r*_*g*_ = −0.47, SE = 0.03, *p*_FDR_ < 0.001), GrimAge (*r*_*g*_ = 0.46, SE = 0.07, *p*_FDR_ < 0.001) and frailty index (*r*_*g*_ = 0.38, SE = 0.03, *p*_FDR_ < 0.001). Similarly, mvAge was genetically correlated with all substance use traits except cannabis ever use, with particularly strong associations with mvSUD, tobacco use disorder (*r*_*g*_ = −0.47, SE = 0.03, *p*_FDR_ < 0.001), smoking initiation (*r*_*g*_ = −0.43, SE = 0.02, *p*_FDR_ < 0.001), cannabis use disorder (*r*_*g*_ = −0.39, SE = 0.04, *p*_FDR_ < 0.001) and problematic alcohol use (*r*_*g*_ = −0.31, SE = 0.02, *p*_FDR_ < 0.001). Note that many of these correlation coefficients are negative, indicating that the same genetic factors increasing the genetic liability for one trait are associated with a decreased liability for the second trait [39]; for the ageing traits that capture healthy (rather than accelerated) ageing, any negative genetic correlations between them and the substance use measures thus reflect increased genetic liability for substance use being associated with decreased liability for healthy ageing and vice versa.

Among individual substance use/disorder traits, smoking‐ and alcohol‐related phenotypes demonstrated robust genetic correlations with ageing measures. Smoking initiation and tobacco use disorder were both significantly correlated with seven of eight ageing traits, including GrimAge (TUD: *r*_*g*_ = 0.52, SE = 0.07, *p*_FDR_ < 0.001; SI: *r*_*g*_ = 0.55, SE = 0.05, *p*_FDR_ < 0.001), parental lifespan (TUD: *r*_*g*_ = −0.51, SE = 0.03, *p*_FDR_ < 0.001; SI: *r*_*g*_ = −0.40, SE = 0.02, *p*_FDR_ < 0.001) and healthspan (TUD: *r*_*g*_ = −0.36, SE = 0.04, *p*_FDR_ < 0.001; SI: *r*_*g*_ = −0.33, SE = 0.03, *p*_FDR_ < 0.001). Similarly, problematic alcohol use and drinks per week exhibited significant genetic correlations with seven and six ageing traits, respectively, including GrimAge (PAU: *r*_*g*_ = 0.36, SE = 0.05, *p*_FDR_ < 0.001; DPW: *r*_*g*_ = 0.27, SE = 0.05, *p*_FDR_ < 0.001), parental lifespan (PAU: *r*_*g*_ = −0.33, SE = 0.03, *p*_FDR_ < 0.001; DPW: *r*_*g*_ = −0.15, SE = 0.03, *p*_FDR_ < 0.001) and mvAge (PAU: *r*_*g*_ = −0.31, SE = 0.02, *p*_FDR_ < 0.001; DPW: *r*_*g*_ = −0.10, SE = 0.02, *p*_FDR_ < 0.001). Notably, although the genetic correlations for drinks per week were consistently weaker than those for problematic alcohol use, Steiger's *Z*‐test analyses—used for comparing correlations among nonindependent groups—revealed no significant differences between the two sets of associations (all *p* > 0.05).

### 3.2 Mendelian Randomization

**FIGURE 3:**
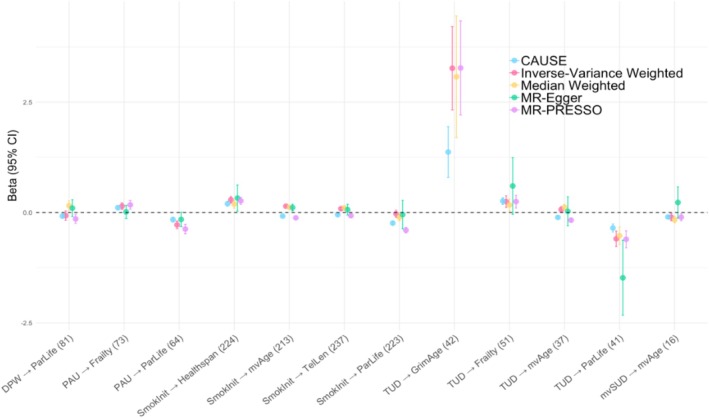
Effect size estimates for trait pairs that were significant in three or more MR tests based on an FDR‐corrected *p*‐value threshold. Exposures include tobacco use disorder (TUD), smoking initiation (SmokInit), problematic alcohol use (PAU), drinks per week (DPW) and a multivariate substance use factor (mvSUD). Outcomes include GrimAge, healthspan, Frailty Index, telomere length (TelLen), parental lifespan (ParLife) and a multivariate ageing factor (mvAge). The number of SNPs used in the MR test for each pair is shown in parentheses. As shown, results from each approach are largely in agreement with one another, with the error bars (representing 95% confidence intervals) generally overlapping with one another across methods.

For each of the significantly genetically correlated trait pairs, we evaluated evidence for causation using IVW‐MR, median weighted MR, MR‐Egger, MR‐PRESSO and CAUSE. Despite their differing assumptions, these methods largely showed agreement with one another, with widespread evidence of causality (Figure 3 and Tables S4–S9). Across the 44 substance use → ageing relationships examined, 32 showed significant evidence of causal effects in at least one of our MR approaches after multiple testing correction.

IVW‐MR, our primary analysis found significant effects of cannabis use disorder, problematic alcohol use, smoking initiation and tobacco use disorder on various ageing metrics (Table S4). Specifically, IVW‐MR found significant causal effects of smoking initiation on mvAge (*β* = 0.14, SE = 0.01, *p*_FDR_ < 0.001), as well as significant causal effects of tobacco use disorder on parental lifespan (*β* = −0.59, SE = 0.09, *p*_FDR_ < 0.001)—two findings that were supported by all four of the other MR approaches. Similarly, all MR methods except MR‐Egger supported the IVW‐MR findings of a significant effect of smoking initiation on healthspan (*β* = 0.29, SE = 0.04, *p*_FDR_ < 0.001) and telomere length (*β* = 0.09, SE = 0.02, *p*_FDR_ < 0.001), as well as that of tobacco use disorder on GrimAge (*β* = 3.37, SE = 0.48, *p*_FDR_ < 0.001). Finally, although IVW‐MR found a significant effect of cannabis use disorder on parental lifespan (*β* = −0.16, SE = 0.03, *p*_FDR_ < 0.001) and mvAge (*β* = 0.05, SE = 0.01, *p*_FDR_ < 0.001), these relationships were not replicated in our CAUSE analyses and lacked sufficient power to be tested in any of our other MR methods.

To further clarify the mechanisms underlying these results, several sensitivity analyses were also conducted. First, the MR‐Egger intercept (i.e., the *I* ^2^ statistic) did not differ from zero for any of the significant IVW‐MR tests (all *p* > 0.05), indicating that the MR assumption of no directional pleiotropy is being met (Table S9). Although Cochran's *Q—*which serves as an indicator of heterogeneous effects among our genetic instruments—was significant across nearly all significant IVW‐MR results, our IVW‐MR findings persisted after leave‐one‐out analyses (Table S10), suggesting no single variant was disproportionately influencing results. We similarly found no significant effects in our MR‐PRESSO distortion tests (Table S5), providing evidence that our causal effects are not being driven by outliers. Finally, there was little evidence of horizontal pleiotropy, with all IVW‐MR significant trait pairs except tobacco use disorder → GrimAge (an expected finding, as GrimAge is trained on methylation markers for cigarette smoking) being insignificant in our MR‐PRESSO global tests.

Lastly, we also examined evidence of reverse causality, testing the effects of ageing on substance use for each of our significantly genetically correlated trait pairs (Tables S4–S9). Across the 42 sufficiently powered trait pairs, only one (parental lifespan → smoking initiation) showed a significant effect in any of our MR tests. However, this relationship was only significant in median weighted MR and also produced a significant result in the MR‐PRESSO distortion test, indicating that it may be driven by the effects of pleiotropic outliers.

## 4 Discussion

Prior research has consistently linked substance use to adverse ageing outcomes, with observational studies finding associations between substance use and mortality [40], healthspan [41], frailty [42], telomere length [43, 44] and epigenetic age [45, 46]. However, the extent to which these relationships reflect causal effects, shared genetic influences or unmeasured confounding has remained unclear. In the present study, we sought to build upon this literature by examining the relationships between substance use/disorders—including tobacco, alcohol, opioid, and cannabis use and use disorders—and a variety of ageing markers using measured genomic data. Using Linkage Disequilibrium Score Regression (LDSC), we found widespread evidence of genetic correlations between substance use behaviours and ageing indices. Notably, despite cannabis use disorder correlating with nearly all ageing metrics, lifetime cannabis ever use was only correlated with parental lifespan, supporting previous findings that casual or experimental cannabis use may not have the same genetic aetiology or long‐term consequences as more problematic patterns of use [47, 48]. Furthermore, in contrast to prior literature showing an association between substance use (particularly alcohol and tobacco) and dementia [49, 50], Alzheimer disease showed few significant correlations with our substance use traits. Although this may be indicative of Alzheimer's disease and substance use having distinct genetic architectures, we cannot rule out the possibility that these null findings were instead driven by the Alzheimer GWAS' relatively small effective sample size or their inclusion of adult offspring of individuals with dementia/Alzheimer's as ‘proxy’ cases [28].

For each significantly genetically correlated trait pair, we implemented several MR approaches to examine the extent to which these correlations are being driven by the causal effect of substance use on ageing. Across our MR approaches, we found consistent evidence that the genetic predispositions to smoking initiation and tobacco use disorder have a causal effect on lifespan, health and markers of biological youth; we similarly found an effect of genetic predispositions to problematic alcohol use and cannabis use disorder on our ageing metrics, though these effects (particularly those of cannabis use disorder) were less consistent across methods. Despite it having consistently high genetic correlations with our ageing traits, the genetic liability for the multivariate substance use disorder factor (mvSUD) did not show any significant causal effects on our ageing indices. While surprising, such a finding may indicate that involvement with specific substances, rather than a predisposition to general disordered substance use, may be driving these SUD‐ageing associations. Finally, we found scant evidence of reverse causality (i.e., ageing impacting substance use) suggesting that the reported increase [2] in SUD rates among older adults is not necessarily occurring in response to the physical and mental challenges of ageing—at least as captured by our indices—as has been previously theorized [51].

Although our analyses focused on direct genetic effects, it is important to acknowledge that environmental and lifestyle factors, such as diet, physical activity and social engagement, play a critical role in ageing [52] as well as some aspects of substance use. A common contributor to these factors is socioeconomic status (SES), which may mediate some of the observed causal relationships. For example, there is evidence [53, 54, 55] that the increased access to healthcare afforded to individuals of higher socioeconomic status may mitigate the negative consequences of substance use, whereas those from disadvantaged backgrounds may experience compounding risks that ultimately accelerate ageing; this is particularly problematic for studies whose population‐based cohorts have a ‘healthy volunteer’ bias, in which genetic correlations between SES, substance use and other mental/physical health conditions (all of which are associated with accelerated ageing) are known to exist [56, 57]. Such factors could introduce unmeasured confounding in our MR analyses, potentially biasing causal estimates if genetic proxies for substance use also capture broader lifestyle differences linked to ageing outcomes. Moreover, our findings of causal effects of substance use behaviors on parental lifespan highlight the potential for intergenerational influences, where familial clustering of health behaviors, socioeconomic conditions, chronic stress or shared genetic risks could shape both offspring substance use and parental ageing trajectories. Future research integrating genetically informed designs with rich environmental data—including within‐family MR approaches, longitudinal methods, sibling comparisons and studies evaluating ageing in negative control samples (e.g., never smokers or those with nondisordered alcohol consumption)—will be essential for disentangling these complex pathways, triangulating evidence, improving causal inference and identifying modifiable risk factors that could mitigate the adverse effects of substance use on ageing.

In addition to the potential confounding effects of SES and other environmental factors, other caveats remain noteworthy. First, as discussed, MR is an exploratory approach that relies on several strong assumptions, some of which cannot be met—or even tested—for complex polygenic phenotypes [58]. Although we implemented numerous sensitivity and follow‐up analyses to help test these assumptions and mitigate any confounding, we also recognize that these methods cannot fully eliminate potential biases. Thus, causal findings from MR approaches should always be interpreted with caution and further complemented by additional methodological approaches in future research. Second, many of our substance use and ageing summary statistics were derived, at least in part, from the UK Biobank. This serves as an important limitation, both because the UK Biobank suffers from known volunteer bias [59, 60] and because utilizing substance use and ageing phenotypes from the same cohort could induce potential biases due to overlapping genetic effects. Although statistical corrections were applied in the present study, some residual confounding cannot fully be ruled out. Third, we cannot exclude the possibility that some portion of these null findings—particularly those involving cannabis, opioids and Alzheimer disease—were due in part to smaller GWAS sample sizes and a consequent lack of power to detect effects, nor can we dismiss the potential consequences of lower rates of recent SUDs in older individuals with health conditions (the ‘sick quitter’ phenomenon), survivorship/selection bias, unmodelled interactive effects or nonrepresentative GWAS samples [61]. Finally, to prevent ancestry/population‐based confounding, all ageing‐related summary statistics were derived from GWAS that only included individuals of European genetic ancestry, limiting the generalizability of these findings to other ancestries. Given that genetic architectures, allele frequencies and environmental exposures can differ across populations, the relationships between substance use and ageing may not be identical in non‐European ancestral groups. Future studies should therefore prioritize multiancestry GWAS to enhance both the robustness and applicability of genetic insights into ageing.

Stigma associated with the acknowledgement of SUDs and the consequent reluctance to query older adults about their substance use hinder effective and tailored interventions in this age group. Collectively, these findings provide insight into the mechanisms underlying the link between substance use and ageing and contribute to a growing body of evidence chronicling the harmful effects of substance involvement—particularly tobacco use—on health and longevity. Additionally, these results underscore the importance of integrating genetic insights into public health strategies to effectively reduce the burden of SUDs across the lifespan.

## Author Contributions

Jared V. Balbona, Ryan Bogdan, Arpana Agrawal and Emma C. Johnson were responsible for the study concept and design and contributed to the interpretation of findings. Jared V. Balbona, Emma C. Johnson, Elliot C. Nelson and Paul Jeffries contributed to data acquisition and analysis. Jared V. Balbona and Emma C. Johnson led the data analysis, visualisation and manuscript drafting with assistance from Aaron J. Gorelik, Elliot C. Nelson, Ryan Bogdan and Arpana Agrawal. All authors critically reviewed the manuscript, provided revisions and approved the final version for publication.

## Conflicts of Interest

The authors declare no conflicts of interest.

## Supporting information


**Table S1.** Summary of methods used, adapted from Burgess et al. (2023).
**Table S2.** Samples used and phenotype definitions.
**Table S3.** LDSC Genetic Correlation Results.
**Table S4.** IVW‐MR with Multiplicative Random Effects Results.
**Table S5.** MR‐PRESSO Results.
**Table S6.** CAUSE ELPD Results.
**Table S7.** CAUSE Median Interval Results.
**Table S8.** Median Weighted Results.
**Table S9.** MR‐Egger Results.
**Table S10.** Leave‐One‐Out Sensitivity Analyses for Significant IVW‐MR Tests.

## Data Availability

The present study utilized publicly available summary statistics for each of our analyses. Sources for each trait can be found in Table S1.
